# Supplementary material for: Lipid packing and cholesterol content regulate membrane wetting and remodeling by biomolecular condensates
Source: Nat Commun. 2025 Mar 20;16:2756. doi: 10.1038/s41467-025-57985-2 (PMC11926106; doi:10.1038/s41467-025-57985-2)
Supplement: Supplementary file 2 — Description of Additional Supplementary Files [file 41467_2025_57985_MOESM2_ESM.pdf]

Description of Additional Supplementary Files

File Name: Supplementary Movie 1

Description: Confocal microscopy z-stack of a giant vesicle composed of DOPC:DPPC 1:1 labeled with 0.1 mol% ATTO 647N-DOPE (green) displaying fluid/gel phase coexistence in contact with a glycinin condensate containing 4 %v/v of FITC labeled protein (magenta) at the working conditions (150 mM NaCl, 23°C, 10mg/mL glycinin). The condensate is only in contact with the fluid phase.

File Name: Supplementary Movie 2

Description: Confocal microscopy z-stack of a giant vesicle composed of DOPC:DPPC:Cholesterol 1:1:1 (green) displaying liquid-disordered/liquid-ordered phase coexistence in contact with a glycinin (magenta) at the working conditions. The condensate is only in contact with the liquid-disordered phase.

File Name: Supplementary Movie 3

Description: Confocal microscopy z-stack of a giant vesicle composed of DOPC:SM:Cholesterol 1:1:1 (green) displaying liquid-disordered/liquid-ordered phase coexistence in contact with a glycinin condensate (magenta) at the working conditions. The condensate is only in contact with the liquid-disordered phase.

File Name: Supplementary Movie 4

Description: Confocal microscopy z-stack of a giant vesicle composed of DOPC (green) in contact with a glycinin condensate (magenta) at the working conditions. Tubes are formed at the membrane-condensate interface protruding into the condensate phase.

File Name: Supplementary Movie 5

Description: Confocal microscopy z-stack of a giant vesicle composed of DOPC:DPPC 1:1 (green) in contact with a glycinin condensate (magenta) at the working conditions. Tubes are formed at the membrane-condensate interface protruding into the condensate phase.

File Name: Supplementary Movie 6

Description: STED microscopy z-stack of the membrane channel of the condensate-membrane interface for a giant vesicle composed of DOPC in contact with a glycinin condensate at the working conditions. Tubes formed at the interface are resolved, see Figure 5D for tube diameter values.

File Name: Supplementary Movie 7

Description: STED microscopy z-stack of the membrane channel of the condensate-membrane interface for a giant vesicle composed of DOPC:DPPC 1:1 in contact with a glycinin condensate (magenta) at the working conditions. Tubes formed at the interface are resolved, see Figure 5D for tube diameter values.

File Name: Supplementary Movie 8

Description: Confocal microscopy z-stack of the membrane channel (green) of a giant vesicle composed of DOPC in contact with a glycinin solution at 365 mM NaCl. Nanotubes are adhered to the outer membrane surface and a double-membrane sheet is observed at one of the vesicle poles.
